# Toward Data-Informed Care in Long-Term Care: Qualitative Analysis

**DOI:** 10.2196/69423

**Published:** 2025-09-12

**Authors:** Suleyman Bouchmal, Katya YJ Sion, Jan PH Hamers, Sil Aarts

**Affiliations:** 1 Department of Health Services Research, Limburg Living Lab in Ageing and Long-Term Care, Maastricht University, Duboisdomein 30, Maastricht, 6229 GT, The Netherlands, 31 433882222; 2 MeanderGroep Zuid-Limburg, Landgraaf, The Netherlands

**Keywords:** long-term care, data, cocreation, data-informed, quality of care

## Abstract

**Background:**

In long-term care (LTC) for older adults, data on client, employee, and organization levels are collected in various ways, covering quality of care, life, and work. There is, however, a lack of understanding of how to introduce data-informed care in LTC and thus create value from data.

**Objective:**

This study aims to investigate the experiences and perceptions of various stakeholders in LTC regarding data and data-informed care.

**Methods:**

A qualitative study using the World Café cocreation technique was conducted with a diverse group of LTC stakeholders. Four questions were addressed: (1) What thoughts do you have when you hear the term “data” in relation to LTC? (2) What purposes do data have (in the future) in LTC? (3) What knowledge and skills are needed to enable data-informed care? (4) How can data contribute to and improve multidisciplinary learning? Stakeholders’ notes and the plenary summary were analyzed using conventional content analysis.

**Results:**

Stakeholders included nurses, members of client councils, data specialists, researchers, and managers (N=20; mean age 50, SD 13 years). Five themes were identified: (1) despite uncertainty, the benefits of using data outweigh the associated risks; (2) the lack of accessibility and uniformity hinders integrating data-informed care; (3) human resources and finance departments pioneer data usage; however, potential lies in clinical decision-making; (4) data-informed care demands individual, collective, and organizational prerequisites; and (5) multidisciplinary collaboration enriches collective knowledge regarding data.

**Conclusions:**

Introducing data-informed care requires enhancing data literacy of health care professionals, establishing clear communication about the role of data within the organization, and introducing new job positions, such as data scientists. Data-informed care was considered a multidisciplinary approach in which data have a supportive role to enhance collective understanding and are considered crucial for improving quality of care.

## Introduction

Data are crucial for making the right decisions in health care systems [1]. Information systems and electronic health records (EHRs) improve data management, offering potential benefits, such as enhancing the quality of care and increasing the knowledge levels of health care professionals [2,3]. For example, in clinical decisions, prompt data entry allows for timely access to information and better decision-making [4,5]. Additional examples include the facilitation of information-sharing, which enables collaborative approaches to decision-making [6,7].

In long-term care (LTC) for older adults, vast amounts of data are consistently collected on client, employee, and organizational levels [8,9]. Data can be collected in various ways, such as conversations, questionnaires, and the use of technologies like sensors or wearables. These data can, for example, provide new insights regarding quality of care, support the early detection of diseases, and enhance decision-making [10,11]. Despite the potential of data, integrating data into daily care practice is challenging due to uncertainties about interoperability regarding different data sources (ie, EHRs) and how to use this data for clinical decision-making [12-14]. In addition, health care professionals are often ambivalent about data and the use of information technology [15,16].

Clients in LTC often have complex and multifaceted needs that require, besides clinical data, additional considerations, such as personal preferences [17,18]. Consequently, challenges arise in decision-making processes due to the context, available data, and characteristics of the decision-makers, such as experience and skills [19]. Previous research has argued that delivering care to individuals with complex care needs should be of high value, team-based, and data-informed [20,21]. Therefore, the informative role of data to support several processes, including decision-making and care delivery, is gaining increased attention in LTC [22-24]. While data-driven methods prioritize decisions based solely on information, data-informed care balances quantitative data with valuable insights from previous experiences and qualitative input from clients, relatives, and health care professionals [25,26]. As data-informed care has not yet been implemented in LTC, however, this may indicate that LTC clients are not receiving the most appropriate care [27].

To date, there is a lack of understanding of how data-informed care can be introduced in LTC settings. For example, the literature regarding the implications of data-informed care for different stakeholders (eg, caregivers, managers, nurses, and data specialists) is scarce [28,29]. Moreover, it remains uncertain which specific requirements are necessary and how LTC organizations and their stakeholders view the role of data in LTC [30]. Therefore, this study aims to investigate the experiences and perceptions of various stakeholders in LTC settings regarding data and data-informed care practices. The findings in this study provide insights into the status quo of data usage and highlight topics to transform toward data-informed care practices in LTC.

## Methods

### Design

This qualitative study used the cocreation method “The World Café,” which allows an open conversation with a variety of stakeholders [31]. This creative method establishes informal discussions in small groups about central questions [32]. The chosen methodology aligns well with the exploratory aims of this study, making it a suitable approach for gaining initial insights [33]. In 3 constructive rounds, collaborative knowledge was created with the stakeholders and evaluated in a plenary session in which the findings were summarized and presented to the group for reflection and feedback [31].

### Participants

This study was embedded within the interdisciplinary core group “Data Science of the Limburg Living Lab in Aging and Long-Term Care” (in Dutch: Academische Werkplaats Ouderenzorg Limburg [AWO-L]) [34]. This formal multidisciplinary network consists of a collaboration among Maastricht University, 9 large LTC organizations, and 3 educational institutions (secondary, vocational, bachelor’s, or master’s level). Together, the Limburg Living Lab delivers care to more than 50,000 clients daily with the support of 27,000 employees. The mission of the network is to conduct scientific research to contribute to the improvement of the quality of life for older adults and their relatives, quality of care, and quality of work for individuals active in LTC [34].

For this study, all organizations within the AWO-L Living Lab were approached and invited to participate based on purposive sampling. This study aimed to include a diverse group of stakeholders. In total, 3 LTC organizations and 2 educational institutes were registered. The principal investigator provided an informative presentation on location. Interested individuals were invited via an email in which the objectives of the study and practical information about the World Café sessions were provided, including the date, location, and time. For those who were interested in participating, a link was included in the email to register. Stakeholders were not required to have prior knowledge of data or data-informed care but were expected to be familiar with the LTC context—either through direct care provision for older adults or through involvement in research or education related to this setting. At the start of the World Café session, a presentation was delivered to introduce the concept of data-informed care. This allowed stakeholders to develop a common interpretation before engaging in group discussions.

### Data Collection

Data collection took place in January 2024 using the seven design principles for conducting a World Café session: (1) set the context, (2) create a hospitable environment, (3) explore questions that matter, (4) encourage everyone’s contribution, (5) connect diverse perspectives, (6) listen together for patterns and insights, and (7) share collective discoveries [35].

Starting the cocreation session, the stakeholders were welcomed with an introductory presentation to review the objectives of the World Café as well as an introduction into the concept of data-informed care. Time was allocated for questions from the stakeholders regarding the information provided by email or during the presentation. Thereafter, stakeholders received an informed consent form that detailed important aspects of the session. This included the type of information that was collected (ie, photos during the session, audio recordings of the plenary summary, and written answers given by the stakeholders), and how this information would be analyzed, processed, and stored. After signing the informed consent, the stakeholders were given a small toolkit containing Post-its, a notepad, markers, and pencils to provide written answers during the World Café session.

The World Café session was hosted in a public venue that simulated a restaurant’s layout, with 4 round tables, as well as snacks and beverages. The World Café method is described as being conducted in a café-style layout of tables, which encourages enthusiasm, contribution, and participation [31,36]. Hence, the World Café facilitates an open conversation with a variety of stakeholders on several topics. The stakeholders were placed into 4 heterogeneous groups based on their profession and organization. Each group gathered at 1 table, and the group’s composition remained unchanged during the cocreation session. In total, 4 tables (and thus 4 groups) were established [35]. All tables were hosted by a “moderator”: a member of the research team with experience in qualitative data collection and in-depth knowledge about the research topic.

For each table, 1 central question and prompts were formulated during 3 meetings prior to the World Café with the research team, based on their relevance to the aim of this study (Table 1) [37]. All moderators participated in a preparatory meeting prior to the World Café to collaboratively discuss the questions and prompts aligned with the study’s aim. Moderators also received additional information on the session’s structure, along with a written manual detailing the prompts assigned to each table. The prompts, which were subquestions regarding the central questions, were used to guide and enhance dialog to acquire a collective understanding.

**Table 1.** Central question per table.

Central questions	Examples prompts
T1 - What thoughts do you have when you hear the word “data” in relation to LTC^a^?	Why do we collect data in LTC? What information does a health care professional need to provide care? What objection do you have to using data in the care process?
T2 - What purposes will data have (in the future) in LTC?	Could data support improve the quality of care? Why should a health care professional consider data usage in their work? What role has data regarding decision-making?
T3 - What knowledge and skills are needed to enable data-informed care?	How can data enhance care practices? What competencies are required regarding data-informed care? What systems or other resources does the health care professional need here?
T4 - How can data contribute to and improve multidisciplinary learning?’	What is multidisciplinary working, and what role has learning and improving played in this? How can multidisciplinary learning support care practices? What challenges should be considered when integrating data into multidisciplinary learning?

a LTC: long-term care.

Each group discussed the corresponding central question for 30 minutes. In the first round, the stakeholders introduced themselves within their group. As the composition of the groups remained unchanged, introducing oneself once was sufficient [35]. After the moderators stated the central question, stakeholders started brainstorming individually and wrote their ideas down on Post-its before starting the conversation, ensuring that all stakeholders’ perspectives were included [38]. Afterward, the dialog between the stakeholders started, and new ideas and answers were formulated on Post-its. The collaborative nature of the World Café methodology aligns with key principles of participatory research by fostering inclusive discussion and meaningful stakeholder engagement. In particular, it facilitates coconstruction, a higher level of participation as defined in adaptations of the participation ladder, where stakeholders actively contribute to shaping insights rather than functioning merely as data sources [39].

In the second and third rounds, the groups shifted to new tables. The moderators started by summarizing the insights of the previous rounds to the new group via the Post-its from the latter group. This allowed stakeholders to link with the previous group and build upon their work to discover new insights regarding the central question [40]. The moderators then stated the central question and let the stakeholders brainstorm individually again before starting the conversation. By expanding on each other’s thoughts, the World Café sessions allowed “cross-pollination,” meaning that sharing and interchanging information and thoughts enriches the group’s capabilities and connects diverse perspectives, resulting in collective knowledge [31]. To conclude the 3 rounds, the 4 moderators provided a plenary summary of the overall findings from their tables. Additional comments or feedback shared during the plenary reporting of each table’s insights were incorporated into the results. All stakeholders present during the world-café session were given the opportunity to share their opinion or provide comments based on the plenary reporting, in case something was missing or misinterpreted.

### Data Analysis

The informed consent acquired additional information from each stakeholder, namely their age, gender, the name of their organization, their job title, and their years of work experience in their current profession, as described in the “Results” section. Descriptive analysis was performed to provide insights into the demographics of the stakeholders (eg, mean age, SDs, and range).

The notes on the Post-its and the written text on the sheet from the tables, as well as the audio record of the plenary summary, were transcribed verbatim [41]. The transcripts (ie, notes per table and the plenary summary) were subsequently uploaded to the qualitative analysis software MAXQDA (VERBI Software GmbH [42]). The first author familiarized himself with the data by reading all transcripts multiple times, which facilitated an in-depth understanding of the data [43]. Conventional content analysis was conducted to describe the experiences and perceptions of stakeholders’ daily work with data in LTC [44,45]. Conventional content analysis avoids predetermined categories and inductively processes the data. Therefore, categories or themes, along with their names, emerge from the data when using a systematic approach [46,47]. This study conducted the conventional content analysis by following up with systematic coding [46].

The first author conducted the coding process by performing a line-by-line analysis that identified meaningful units of information [48]. These text segments were “open coded” with overarching labels describing the condensed meaning. The descriptive labels of the open-coded segments were summarized by grouping similar codes to identify clusters of codes. By integrating the codes into clusters, overarching categories were formulated [49]. Establishing overarching categories by specifying subcategories is often referred to as axial coding [50]. This process was evaluated by all coauthors. The content and interpretation of the themes were discussed with the whole research team [51]. The translation process was checked for content and context independently within the research team, based on the central questions, used prompts, original notes written by the stakeholders, and translations conducted by the first author.

### Ethical Considerations

The study protocol was approved by the ethics committee of Maastricht University (approval number: Faculty of Health, Medicine, and Life Sciences - FHML-REC/2024/055). All information about the aim of this study was communicated to the stakeholders in advance by email. Informed consent was provided, and participation was strictly voluntary. Withdrawal from the study was allowed at any moment for any or no reason. To guarantee the anonymity of the stakeholders, no names or organizations were documented.

## Results

### Overview

The descriptive characteristics of the stakeholders are reported in Table 2. In total, 20 stakeholders participated with a mean age of 50 (SD 13) years. The stakeholders had, on average, 16 (SD 9) years of working experience, and 55% (11/20) were female. This study included 7 different professions from LTC organizations and educational institutions.

**Table 2.** Descriptive characteristics of stakeholders (N=20).

Characteristics	Value
Age (years), mean (SD; range)	50 (13; 33-79)
Work experience (years), mean (SD; range)	16 (9; 3-40)
Sex (female), n (%)	11 (55)
Profession, n (%)	
Health care professionals^a^	6 (30)
Business Intelligence specialists^b^	3 (15)
Members client council	3 (15)
Education^c^	4 (20)
Managers	4 (20)

a health care professionals included 4 nurses, 1 specialist in involuntary treatment, and 1 specialist in client well-being.

b Business Intelligence specialists included 2 Business Intelligence architects and 1 specialist in data storage and exchange.

c Education included 2 teachers and 2 researchers.

Five themes were identified during the World Café session [52]: (1) despite uncertainty, the benefits of using data outweigh the associated risks; (2) the lack of accessibility and uniformity hinders integrating data-informed care; (3) human resources and finance departments pioneer data usage, yet potential lies in clinical decision-making; (4) data-informed care demands individual, collective, and organizational prerequisites; and (5) multidisciplinary collaboration enriches collective knowledge regarding data. The 5 themes, including their underlying axial codes and some examples of direct quotations in stakeholders’ notes, are reported in Table 3.

**Table 3.** Overview of themes, axial codes, and stakeholders’ notes.

Themes and axial codes	Stakeholders’ notes (direct quotations)
Despite uncertainty, the benefits of using data outweigh the associated risks	
Knowledge about data	Data is just a word–data is not used (yet)–there is a lot of data in LTC.
Negative feelings	Data collection should not become the aim–fear of the unknown–risk that data steers a professional too much.
Current applications	AI in practice–eHealth (data that is not necessarily added or used by humans)–going from retrospective to real-time insights, to prospective improvements.
Ethical dilemmas	Uncertainty about reliability of data-Ethical dilemmas about data usage.
Data’s potential for care	Data usage of quality of care–data supports tailored care to the client–identifying group selections.
The lack of accessibility and uniformity hinders integrating data-informed care	
Indicating variety of data	Demographical information – Clinical Measurements – kinds of data, including audio, video, qualitative and quantitative.
Uniformity in systems	Standardizing definitions in the systems—System often requires jargon, should switch to layman’s’ language—database structure is crucial for enhancing uniformity.
Simplification	Translating data to present understandable information for clients, informal caregivers and public–professionals will be supported if data becomes more comprehensive and easy to use–simplifying data improves multidisciplinary learning and improving.
Interoperability	Sharing data and associated solutions to increase data usage–LTC can continuously improve by connecting data between systems–Link external sources and strive for open data.
Electronic health record	Double checks in EHR–Data could support medication registration–Compliance cannot be tracked with data yet.
Information gain	Systems should be unambiguously and easy to use–Making data both findable and usable–With LTC, agreements are needed for accessibility and governance of data.
Accessibility	Creating overview of aging population (in region)–Matching eHealth and specific client(characteristics)–Insight into data of future clients.
Centralizing storage	Working towards one platform and format for different disciplines-Centralizing information–Reducing workload or pressure as data entry and retrieval are in one place.
HR and finance departments pioneer data usage, yet the potential lies in clinical decision-making	
Organizational opportunities	Determination of long-term vision, supported by financial numbers–Organize care differently (more care with less resources–Data can be used positively or negatively to finance care.
Data collection and analysis techniques	Collecting information on different levels, to gain insights what is important and for who–classifying systematic problem analysis–Identifying the different instruments for data collection will result in less administration costs.
Predictions	Data can support in findings patterns and trends–Estimating lifestyle and preferences of clients–Predicting care intensity.
Data-informed care demands individual, collective, and organizational prerequisites	
Education, training, and support	Education to understand the goal of data and information–Support needed for individuals with less digital literacy–Education or train employees systematically.
Teamwork and collective skills	Communicational skills within a team–Dare to innovate-mentality needed in a team–Every team should include an attention raiser or data specialist.
Individual contribution toward data-informed working	Feeling the urge to do own contribution independently or together with informal caregiver)–Taking control and responsibility–Understanding that data should support and not influence.
Requirements for learning and improving	Multidisciplinary collaboration with different layers (care, cure, management, IT)–Organizational or team objective should be formulated and communicated more clearly with everyone–Define responsibilities and roles.
Multidisciplinary collaboration enriches collective knowledge regarding data	
Central role for data	Data supports individuals in multidisciplinary approaches with shared and offering new ideas–Adhering to Evidence Based Practice working principles–Enhance dialogue and understanding between different professions.
Collaborative and shared decision-making	Integrating multiple sources and professions allows for better consideration and substantiation–Appropriately implementing SDM result in more, better dialogue with clients–making decisions as a team.
Striving for overarching goals	Teams should mitigate working with limited perspectives from other professions–by working multidisciplinary, the shared objective should be accepted by all–Crucial advantage is “multi-perspectivity” on the one subject.
Collective knowledge	Leaning-by-doing enhances individual digital literacy levels–Gaining knowledge about digital systems, finding information and using that information – Collective understanding acquired by individual contributions.

### Despite Uncertainty, the Benefits of Using Data Outweigh the Associated Risks

When asking stakeholders what data means to them, the majority reported recognizing the presence of “data” in their organizations. They mentioned, however, that it is hard to describe data specifically. In addition, they expressed that data are not part of their daily tasks. As stakeholders discussed their role in collecting data, such as demographic information about their clients, they also indicated being aware of different types of data present in LTC (eg, images, audio, and quantitative).

*Well, initially it was about numbers. Then you have to think of other things that we collect, for example data on wounds or falls or pain and stories in file - stories that employees write down in the files about clients.*

*Information about personnel files but also sensor technology were added – this data was not collected by humans, but collected in a different way, such as video, audio.*

As the stakeholders considered data usage beneficial, they proposed that LTC should start shifting on the organizational level to increase the use of data. Stakeholders mentioned that such transitions should be done not only to enhance data usage but also to allow the evaluation of how decision-making is integrated in the organization. They emphasized the benefit of data use for the quality of care and discussed that LTC organizations should go from retrospective improvements to real-time insights and even to prospective improvements in the future. Several solutions were mentioned by the stakeholders to support such data transitions, for example, artificial intelligence. However, stakeholders noted that the use of data in practice entails both advantages and disadvantages due to underlying uncertainties, including ethical considerations and client privacy concerns.

*First, we need to make sure that shared decision-making really happens. If that is not the case, how do you want to add the data part to introduce data-informed decision-making? Than we should think about the process, before adding data into it. Especially about how we want to offer it to that group.*

[Client, relatives, and healthcare provider]

*Implementation of data into solutions that can mimic ChatGPT in which insights from different disciplines can be linked together. Artificial intelligence may support care processes but could also bring more improvements over time. Everybody in the group [client, relatives, and healthcare provider] could experience then an added value.*

While summarizing the variety of data sources, stakeholders mentioned the negative aspects of using data. For example, a few stakeholders mentioned applications that could automatically collect (real-time) data (eg, sensors) but discussed their concern about the reliability of the data. Moreover, stakeholders indicated that using data for data-informed care includes the risk that health care professionals will be influenced too much by the data without assessing or recognizing their quality.

*Again, not too critical, but critical enough to evaluate the data to see what do the data say, is it correct and does that match my gut feeling? Is that also how I see the client?*

The majority of the stakeholders indicated that using data could support gaining more insights into their clients. They wished for a supportive role for data to oversee their clients, including underlying data that might help them provide care. In addition, care processes on both an individual level (delivery-receiving care) and an organizational level (arranging and organizing care) can experience the benefits of using data, according to the stakeholders. They mentioned that data in LTC help deliver more personalized care and improve quality of care in the organization.

*Because we want to improve the quality of care, we want to be able to get care tailored to the client by providing personalized care. Therefore, we will get a better picture of: OK, we now have this client in a group or subgroup with related data, for example, in a nursing home.*

### The Lack of Accessibility and Uniformity Hinders Integrating Data-Informed Care

A few stakeholders indicated that work processes were established for data entry and retrieval in their systems. Most stakeholders, however, mentioned that the systems in place did not complement their work processes and therefore negatively influenced their daily work. For example, stakeholders mentioned that they noticed differences in data entry between colleagues, mainly due to missing definitions or badly defined processes.

*It is relevant to ensure uniformity by entering data in the same way in an organization. One person puts a “V” for woman (in Dutch: vrouw), and another tries the whole word* “*woman” or another enters an “f” for “female” in English.*

*Another example is weighing our clients. Do we need to weigh our clients with or without clothes: there’s already a difference there.*

The dialog of the stakeholders shifted to the shortcomings in the digital systems. They mentioned that data systems often require jargon, as decided by the developers. Stakeholders wished to switch to a more “layman’s language” approach, as this would support professionals in translating information to make it more comprehensible to their clients, informal caregivers, and even the public.

*The fact is that we are looking for clarity and uniformity, but at the same time you must be able to report in your own jargon. So ideally, we also use systems or even technology that can translate the jargon to everyone’s needs.*

Stakeholders noted that simplifying data presentation was only part of the solution. They also experienced a degree of ambiguity regarding the systems, with the main concern being the lack of central storage. Many recognized that entering data into different systems (eg, EHRs and personal files) increased their administrative burden. Centralizing information on 1 platform in a consistent format could, according to the stakeholders, reduce their workload and enhance data accessibility.

*We would have a lot of new possibilities with developments in the organizations, such as gathering all information at one place–as this would allow you to plan much better, control better what has been said, and monitor more.*

*When it comes to potential, you can also think that more data together has a signaling function. Monitors, signals, help you to say at the right moments: Yes, now, we have to be careful [regarding the situation of this client] and we have to take action now.*

Supposing that the uniformity and accessibility were deemed sufficient, stakeholders mentioned that connecting with other systems would be desirable. Stakeholders indicated that most of their clients were admitted from various organizations, such as hospitals or rehabilitation centers. As these organizations might use different systems, the connections needed to retrieve clients’ data (eg, background and history) are often missing.

*But in specific terms, this is the most important thing we find with this client, this is the most important data that comes with it. Also between other domains, such as primary care, general practitioners, and hospitals, everyone does things in their own way, and that does not connect well with each other.*

*For instance, this is how it works in practice now: You go to the dentist with a cavity, and afterwards you are being referred to a dental surgeon. You will hear probably that they should take another X-ray, because they cannot get the picture taken at the dentist practice, so twice the cost, twice the time, which becomes your concern.*

### Human Resources and Finance Departments Pioneer Data Usage, Yet Potential Lies in Clinical Decision-Making

Stakeholders noted that data are collected at all levels of LTC (eg, the client, department, and organization levels), each using different instruments and having various goals. They mentioned that, although data are not used for clinical decision-making, departments, such as human resources (HR) and finance, are already integrating data insights (eg, resource allocation and bed occupancy per department). Stakeholders observed, however, that data usage in LTC primarily focuses on HR and finance. These departments are supporting the organizations, and insights from these departments are crucial for determining the organization’s long-term vision, sometimes supported by financial numbers. They pointed out that, by using systematic approaches (eg, problem analysis and running reports), these departments could be an example for other departments to show how information should be presented to a wider audience.

*There are also different levels that you have to think about: you have the individual, but you can also learn from each other in the department, and you can learn from each other within the organization.*

*Data usage can be very effective and works for many people. You can show the performance of your staff, and you will consider more the allocation of resources, which is good for the sustainability of healthcare in the future.*

*For example, making an order, how to finance things, a lot can be automated based on data if we can properly see what data we already have.*

Regarding data-collection and -analysis techniques, stakeholders mentioned the importance of gathering information at different levels to understand what is important and for whom. For example, stakeholders mentioned that working in a systematic order and using a limited number of different instruments for data collection could be used to reduce costs—both the financial cost and the burden experienced by health care providers.

*We often see that we want to reduce the administrative tasks for an employee–which of course may lead to lower costs for the organization as a whole.*

*Electronic health records of the person is a system in which a part of the wishes and needs is often missing and is not actually discussed when we look at how we can provide care for this person. Therefore, sometimes preparations in analyzing data could actually accelerate [the care] process.*

Stakeholders also discussed the predictive capabilities of data, noting that it can help find patterns and trends, estimate clients’ lifestyles and preferences, and predict care intensities. They acknowledged that the data collected and analyzed could support care processes.

*For example, if there is a client with behavioral issues, you hope to match them with a care professionals with the necessary expertise–and in a later stadium, you even want to predict to development of the behavioral issues among clients. Data servers than as a signaling function, where it monitors with you together and alerts you at the right moment.*

### Data-Informed Care Demands Individual, Collective, and Organizational Prerequisites

To realize the benefits of using data for decision-making in LTC, stakeholders indicated that several transformations should be considered. First, stakeholders noted a need for training or education to understand data and its potential on an employee level. They indicated that the purpose of data collection is often unclear, as they experienced collecting data as a routine process. Stakeholders mentioned that the lack of clarity in work processes was leading to the underuse of data to provide tailored care or engage clients in dialog—both crucial for decision-making. Stakeholders mentioned that support would be needed for employees with less digital literacy.

*Data literacy, learning the language, digital skills, are more often expected as basis for professionals, but it should not be considered as a basis [set of skills] and we should understand that some professionals really need support in this. Thinking about a digital coach.*

*We also mentioned that such things should be included more in education. For future healthcare professionals, certain aspects will change, as the professionals we train now are definitely different from those we trained twenty years ago.*

*To analyze data in groups, and perhaps, we should do this also during the education, to see whether we can let people get familiar with data earlier, learning what data are and what can be done with them.*

In addition, stakeholders mentioned the urgency of better top-down communication. According to them, management should clearly formulate objectives to improve data usage and clarify processes for data-informed care. Most stakeholders noted that collaboration across different layers and professions was required. Stakeholders also discussed the need for better-defined responsibilities and roles, as stakeholders described data-informed decision-making as a collaborative approach that includes multiple perspectives and experiences. Clear role definitions enhance clarity and focus on organizational objectives. Stakeholders suggested introducing roles, such as attention-raisers and data specialists, to maintain focus and improve data usage and understanding. The importance of these new roles within the LTC organizations lies in their specific skill sets, which contribute to both the team level (ie, fulfilling the collective need to understand and work with data) and the organizational level by facilitating and promoting the adoption of data-informed care. These roles allow for combining domain-specific knowledge with ICT (information and communication technology) and data expertise, enabling the bridging of the gap toward practical care applications.

*If you are not clear about what your vision is as an organization, if you are not clear on what you want to focus on, how can a healthcare professional incorporate data into his or her work?*

*Very important is the communication, collaboration and also a bit of trust.*

*The question is if it is clear to everyone if we discuss a topic in a group setting? If some people say, that is not clear to me and then you immediately notice that everything that has been discussed may be correct, but that there are a number of concepts that were not communicated the right way.*

Stakeholders also noted the importance of teamwork and collective skills, including communication and a “dare to innovate” mentality. For individual contributions to data-informed work, stakeholders mentioned expecting pressure to contribute to group gatherings, including taking control and responsibility. First, stakeholders stressed the need for collaboration across various layers within an organization (ie, health care professionals, managers, and IT specialists).

*It also requires a bit of openness, a bit of innovative capacity–especially from a diverse group of professionals. Suppose we all want to do something with data-informed working. Who could possibly do that? In fact, everyone should be aware of the importance of this, and we should work together with all layers.*

Second, clear communication of the organization’s objects is important. It should be clear what vision the organization has about data usage.

*You will also need a kind of outcome measure, a result and that addresses the needs of the client, this is our main objective. This should be formulated and communicated in a clear manner.*

Finally, responsibilities and roles should be better defined, as should the tasks of each individual.

*You have to determine who is responsible for what, and what are we going to do–in which data can support to determine who is responsible for what. So you interact with the group get to say to everyone: yes, you had to do that, and you had to do that.*

### Multidisciplinary Collaboration Enriches Collective Knowledge Regarding Data

Stakeholders indicated that data-informed care needs a multidisciplinary approach in which data usage supports collaboration. Multidisciplinary collaboration involves health care professionals from LTC organizations and emphasizes the participation of clients and relatives. They mentioned that, by integrating data, experiences, and knowledge, new ideas could be generated. It was highlighted that acquiring general knowledge on data during multidisciplinary collaborations becomes more relevant when different professions gather. Moreover, they expected a collective understanding of data and client relevance. In addition, stakeholders mentioned that data should have a central role in a multidisciplinary approach or should even be a starting point.

*Together, we mainly look at what the needs and wishes of the person are. What information do the relatives of a client already have? How can we achieve this in a combined intervention, a collaboration within our organization? That should be realized.*

*We have to look more for common ground, where we can find each other, and see what we are doing the same, instead of just looking for the differences. We could go a lot further that way, towards a dream scenario–if this could be done with including data to improve multidisciplinary learning.*

Some stakeholders even mentioned that making decisions as a team allowed for comprehensive substantiation, promoting evidence-based practice and justification for care decisions. Stakeholders also noted that team decisions should not mitigate professional perspectives but rather take advantage of the diverse perspectives that occur in such collaborations.

*When receiving the correct information to translate into data from which you have to choose again: which data are essential? Then you check this [information] multidisciplinary, in dialogue–before and during making a decision.*

*As long as you have a shared goal and everybody is open to exchange [their] insights you can work together. And there are many stakeholders that all play a role to contribute and share their piece of knowledge.*

Stakeholders emphasized that multidisciplinary collaborations could improve individual competencies for all those involved (ie, professionals, clients, and informal caregivers). They noted that it was difficult to specify how and which competencies would be affected but that data perform a supportive role that enriches knowledge and experience—as data could simplify complex information by summarizing or illustrating it graphically. As for clients, some stakeholders mentioned that multidisciplinary approaches may result in better dialogs during consults and improved quality of care when data are available as a central component. Therefore, employees require suitable competencies to introduce data during dialogs with clients.

*Not sure of how exactly that [specific set of competencies] would look like, but it will of course help a lot in the whole process to work with data and enhancing multidisciplinary collaborations.*

*Therefore, we need the right match between good employees, good competencies and a specific client to work with. Then we could have more time to discuss what everybody in the group [staff, informal caregiver, and client] wants.*

## Discussion

### Principal Findings

This study aimed to provide insights into the experiences and perceptions of stakeholders from different LTC organizations (eg, health care professionals, client representatives, data and ICT specialists) regarding data and data-informed care. The findings indicate that these stakeholders are aware of the importance of data in LTC organizations but do not yet use data to their full potential. Stakeholders recognized its benefits, particularly in personalizing and improving care quality, and identified diverse types of data, such as quantitative data from wearables and qualitative data from EHRs or video materials. While engaging with data was not perceived as a regular part of their day-to-day professional lives, several stakeholders did recognize their role in collecting and using data (eg, health care professionals documenting information in EHRs and using these data when caring for clients). Data were primarily used for operational purposes, such as HR and finance departments. However, the integration of data into care decision-making remains limited. Stakeholders highlighted key conditions for improving data-informed care, including enhanced data literacy, clearer communication from leadership, and better-defined roles, responsibilities, and work processes. These conditions span different organizational levels—from individual competencies to system-wide coordination and support.

The findings align with previous literature, which emphasizes that health care professionals recognize the potential of data to enhance personalization and care quality, yet remain concerned about data accuracy and adequacy [53]. While data holds the potential to support a collective understanding of clients’ needs and preferences, data is not yet systematically applied in everyday practice. Studies mentioned various factors that contribute to increased data use, such as clearly defined work processes [54], roles [55], and responsibilities [56,57]. In line with this, the uptake of data-informed care requires improvements in data literacy, clearer organizational communication, and clarifying data definitions [54-57]. Especially, data literacy plays a foundational role in enabling data-informed practices. It encompasses a set of competencies related to data interpretation, quality improvement, and communication within a multidisciplinary context [58,59]. Research indicates that several personal competencies may be crucial for adopting data-informed practices, such as problem-solving, reasoning, or critical thinking [60]. Understanding different levels of data literacy across professions enables tailored training programs, such as eLearnings and digital coaches with job-specific cases, to support self-paced learning and improve adoption [23,61-63]. Furthermore, data-informed care is considered a multidisciplinary approach that creates new insights by integrating data, experiences, and knowledge from different disciplines [64,65]. Introducing new roles (eg, data scientist) might enhance data usage, as they require knowledge and skills to leverage data and generate predictions to uncover new insight related to care provision [56]. Especially when care needs are complex, the demand for the skills that support multidisciplinary approaches is growing, as they are often associated with an improved perceived quality of care [66-68]. In health care, research indicates that health care professionals in multidisciplinary teams continue to face challenges in using ICT systems and, consequently, data [28,69].

To date, there are multiple initiatives within LTC to contribute to data-informed care. For example, Business Intelligence (BI) solutions—consisting of systems and activities to process data into information—are often present in these organizations [70-72]. Such BI solutions allow LTC organizations to develop a platform to centralize data from different systems (eg, HR systems, EHRs, and supply chain systems) as they help streamline data entry and retrieval [73]. However, they have yet to be used to support data-informed care for clients. In this study, all participating organizations employed at least 1 BI specialist who supports management by providing data summarized in reports or dashboards. The use, however, is still mainly focused on support for the HR department, while its potential indicates a broader potential in LTC for clinical relevance and quality of care.

To our knowledge, this study is the first to provide an overview of the experiences and perceptions of a diverse group of stakeholders in LTC regarding the use of data. By conducting a World Café session, collective knowledge about data and data-informed care was collected. As all stakeholders had different backgrounds and years of working experience, multiple perspectives were included in this study [51]. To ensure the trustworthiness (ie, credibility and reliability) of this study, multiple actions were deployed. First, data-source triangulation was apparent, as stakeholders from three different LTC organizations and 2 educational institutes participated [74,75]. Second, to enhance trustworthiness in qualitative research, this study provided a detailed overview of the procedures in the data collection and analysis while having a constant dialog within the research team about the codes [76]. Third, to strengthen the confirmability, the main results of the World Café session were summarized and shared in a plenary, as a member check [76]. However, the results of this study should be viewed in light of some possible limitations. First, the authors adopted a reflexive and practice-informed position throughout the study, acknowledging their active role in shaping the research design, data collection, and interpretation of findings. Given the close collaboration with LTC organizations and the participatory nature of the research, the potential for bias was recognized. One factor might be confirmation bias, where researchers—consciously or unconsciously—may have interpreted the data in ways that align with their expectations or prior experiences within the LTC setting [77]. To mitigate this, multiple strategies were used, including triangulation of data sources and iterative coding with multiple researchers. These measures were intended to enhance the credibility of the findings while remaining sensitive to the context in which the research was embedded. Moreover, the plenary session with all stakeholders also served as a validation mechanism to enhance the credibility and interpretability of the findings and supports clarification of ambiguity. Second, the stakeholders who participated in this study might be more interested in data-informed care than the average stakeholder of LTC organizations [78]. Therefore, it might imply that the current result may not be applicable to all stakeholders in the LTC domain. Finally, to realize an impact within the participating organizations to transform toward data-informed care, more stakeholders should have been participating. It supports the dissemination of information and insights while simultaneously considering more experiences and perceptions.

This study identified several challenges that hinder integrating data into daily LTC practices. Addressing these challenges should be a key focus of future research. When prioritizing the adoption of data-informed care in LTC organizations, gaining insights into the status quo of their own data use might be the first step. For example, by conducting a data maturity assessment, several domains (eg, IT architecture, strategic management, data quality, and employee skills) can be evaluated regarding data usage and data-informed working and support for the transformation toward data-informed practices [79]. Gaining insight into these prerequisites for data-informed care within organizations might enhance the support for the development of strategies to embed data-informed care with primary processes. To realize the potential of data-informed care, LTC organizations must prioritize system interoperability, data quality, and a shared understanding of the data’s purpose. Policies should support digital literacy at all levels and promote clear roles, responsibilities, and outcome-oriented data strategies. Building on recent national initiatives in the Netherlands, the Artificial Intelligence Navigator for LTC is well-positioned to integrate these insights for broader dissemination. Moreover, there is also a need to promote standardization and alignment of data entry processes across LTC organizations nationally. Variations in terminology, documentation practices, and digital systems currently limit the usability and reliability of data. Establishing uniform data entry guidelines and encouraging centralized, accessible information systems could reduce administrative burden, improve data quality, and prepare for real-time monitoring and personalized care planning nationwide.

### Conclusion

Stakeholders in LTC recognize data as essential for personalized care; however, challenges like low standardization and fragmented systems hinder the transitions toward data-informed care. Enhancing data literacy, fostering organizational clarity, and implementing roles, such as data scientists, were considered necessities. Seen as a multidisciplinary approach, data-informed care aims to support collective understanding and inform decision-making, ultimately enriching quality of care by integrating diverse perspectives.

## Abbreviations

AWO-L: Academische Werkplaats Ouderenzorg Limburg
BI: Business Intelligence
EHR: electronic health record
HR: human resources
ICT: information and communication technology
LTC: long-term care
